# The use of glucocorticoid in severe fever with thrombocytopenia syndrome: a retrospective cohort study

**DOI:** 10.3389/fcimb.2024.1419015

**Published:** 2024-08-06

**Authors:** Yuzhang Chen, Huan Wang, Fengqin Zhou, Chunxia Guo

**Affiliations:** ^1^ Department of Endocrinology, Union Hospital, Tongji Medical College, Huazhong University of Science and Technology, Wuhan, Hubei, China; ^2^ Department of Infectious Diseases, Union Hospital, Tongji Medical College, Huazhong University of Science and Technology, Wuhan, Hubei, China

**Keywords:** severe fever with thrombocytopenia syndrome, glucocorticoid, mortality, infection, meta-analysis

## Abstract

**Introduction:**

Severe fever with thrombocytopenia syndrome (SFTS) is prevalent in East Asia. However, the use of glucocorticoids (GCs) in the treatment of SFTS remains controversial.

**Methods:**

In this retrospective cohort study, we collected the data from patients with SFTS at Wuhan Union Hospital to evaluate the effect of GC therapy. Mortality and secondary infections were compared as outcomes. After searching public databases, we also included articles that examined GC use in patients with SFTS for meta-analysis.

**Results:**

Patients treated with GC had higher fatality rates (21.1% vs. 11.9%, respectively; *P*=0.006) and a longer length of stay (10.6 ± 5.1 vs. 9.5 ± 4.2, respectively; *P*=0.033). In cohorts adjusted using propensity score matching and inverse probability of treatment weighting, no significant differences in fatality rates and length of stay were observed. A meta-analysis of 4243 SFTS patient revealed that those treated with GCs had significantly higher mortality (OR=3.46, 95% CI =2.12-5.64, *P*<0.00001) and secondary infection rate (OR=1.97, 95% CI=1.45-2.67, *P*<0.0001).

**Discussion:**

GC should be used cautiously when treating SFTS. No significant differences were identified in terms of mortality and secondary infection rates between patients with SFTS treated with or without GC.

## 1 Introduction

Severe fever with thrombocytopenia syndrome (SFTS) is an emerging infectious disease, caused by a novel bunyavirus known as the SFTS virus (SFTSV), mainly affecting East Asia (Liu et al., 2014). The major clinical characteristics of SFTS include fever, gastrointestinal symptoms, and regional lymphadenopathy; laboratory testing reveals significant thrombocytopenia and leukocytopenia (Yu et al., 2011). There are currently no reliable therapies or vaccines (Takayama-Ito and Saijo, 2020), and some patients with SFTS rapidly develop multiple organ dysfunction syndrome, disseminated intravascular coagulation, septic shock or hemophagocytic syndrome (Kato et al., 2016; Seo et al., 2021), which contribute to a high fatality rate of 5.11% in China (Chen et al., 2022).

An overwhelming cytokine storm is a key factor in determining the progression and severity of SFTS. The SFTSV induces hyperproduction of pro-inflammatory cytokines including tumor necrosis factor-alpha (TNF-α), interleukin (IL)-6, IL-1β through nucleotide-binding oligomerization domain-like receptor protein 3 (NLRP3) or other inflammasome activation (He et al., 2021; Liu et al., 2021). Therefore, in addition to antiviral therapy (such as ribavirin) and symptomatic supportive treatment based on severity, clinicians may also administer glucocorticoids (GCs), intravenous immunoglobulin, or even perform plasma exchange to suppress the inflammatory response (Seo et al., 2021).

For over 50 years, synthetic GCs have been widely used to treat inflammatory diseases owing to their ability to inhibit excessive production and release of proinflammatory molecules including IL-6 and TNF-α, while stimulating the synthesis of anti-inflammatory factors such as IL-10 (Cain and Cidlowski, 2017). Using anti-infective medications in the treatment of infectious diseases has been considered incompatible with the ability of GCs to suppress inflammation, GCs has become increasingly used in recent years to treat severe infectious diseases, such as COVID-19 (Chaudhuri et al., 2021) to prevent severe cytokine storm-related complications.

The most recent Chinese expert consensus (Guang. et al., 2022) stated that GCs are not a component of the recommended standard treatment strategy for SFTS. While this consensus also suggests that GCs can be used to treat patients with SFTS-induced hemophagocytic syndromes, and for critically ill patients with SFTS and systemic inflammatory response syndromes, no standards or recommendations for identifying patients with SFTS who are suitable for GC therapy are included. Moreover, no treatment regimens for optimal clinical efficacy have been established, which may lead to inappropriate use of GCs in the treatment of SFTS, resulting in negative patient outcomes. Some case reports have shown that GCs can successfully treat SFTS complicated with serious and fatal conditions, such as encephalopathy (Kawaguchi et al., 2016; Kim et al., 2016; Nakamura et al., 2018) and hemophagocytic syndrome (Kitao et al., 2016). However, four cohort studies conducted over the past three years in China (Xiong et al., 2022; Wang et al., 2023), Japan (Kawaguchi et al., 2021), and Korea (Jung et al., 2021) have been reported GC therapy may increase mortality and complication rates in patients with SFTS.

Therefore, we aimed to further investigate the effectiveness of GCs in the treatment of SFTS through retrospectively analyzing baseline data, clinical symptoms, laboratory test results, and treatment outcomes in patients with SFTS. Wuhan Union Hospital, one of the largest medical institutions located in the most prevalent SFTSV region in China, was used for data collection. We pooled and meta-analyzed our conclusions with those of a previous retrospective cohort study to obtain more reliable conclusions regarding the role of GC therapy in patients with SFTS.

## 2 Materials and methods

### 2.1 Study design

This retrospective cohort study was conducted at the Department of Infectious Diseases at Wuhan Union Hospital from January 2020 to December 2022. Adult patients with fever and thrombocytopenia were included if they had an appropriate epidemiological history and a positive serum SFTSV Ribonucleic Acid (RNA) quantitative reverse transcriptase-polymerase chain reaction (qRT-PCR) test result. Exclusion criteria comprised patients: (i) who had received long-term or short-term GC therapy outside of the hospital, (ii) with other active acute infections in addition to the SFTSV, (iii) discharged from the hospital within 48 h, and (iv) with missing clinical information.

### 2.2 Clinical data collection

The following clinical data were obtained and documented from the electronic medical records: demographic characteristics, vital signs, hospital stay, clinical symptoms, epidemiological history, and laboratory test results. According to the latest expert opinion in China, SFTS severity is divided into four sub-types based on the extent of organ damage and symptoms: mild, moderate, severe, and critically ill. Patients with mild and moderate types were included in the general/middle group and those with severe and critically ill types formed a severe group (Guang. et al., 2022). We collected the results of the first laboratory test within 48 hours of admission, including SFTSV load, inflammatory indices (high sensitivity C-reactive protein, ferritin, procalcitonin), routine blood tests (red blood cells, white blood cells, platelets, hemoglobin, and monocyte, eosinophil, lymphocyte, and basophil levels), liver function (aspartate transaminase, alanine transaminase, total-value bilirubin, direct bilirubin), renal function (creatinine, blood urea nitrogen), and cardiac myosin (cardiac troponin, creatinine-kinase MB). In terms of clinical symptoms, bleeding tendencies mainly manifest as mucosal hemorrhage (e.g., gastrointestinal bleeding and oral mucosal bleeding) and/or disseminated intravascular coagulation. Gastrointestinal symptoms include a lack of appetite, nausea, vomiting, and diarrhea.

The observed outcomes of this retrospective cohort study were length of stay, in-hospital mortality, and secondary infection rate. Secondary infections were defined as new microbial infections that appeared ≥48 h after admission. Patients were considered to have been treated with GCs if they received GC therapy during hospitalization. Most patients received dexamethasone for GC therapy, with a smaller number receiving treatment with hydro-prednisone or methylprednisolone.

### 2.3 Statistical analysis

One-to-one propensity score matching (PSM) was performed strictly using the nearest neighbor algorithm to balance confounding variables between the GC and non-GC groups. PSM analysis using the matching package in R software (version 3.4) generated a balanced SFTS cohort, including the GC group (N=180) and the non-GC group (N=180, referred to matched cohort). To further reduce the potential imbalance between the GC and non-GC groups, we used the inverse probability of treatment weighting (IPTW) algorithm (referred to as the weighted cohort). The same propensity-matching and inverse propensity weighting methods were used for all sub-analyses.

R (version 3.4) and SPSS (version 18.0) software were used to perform all the statistical analyses. Results were considered statistically significant when a two-sided *P*-value was less than 0.05. Enumeration indices such as sex are shown as the number of cases with percentages. Comparison between the GC and non-GC groups was performed using the Chi-square or Fisher’s exact tests. Continuous data are expressed as mean ± standard deviation (SD) or median [inter-quartile range] for normally distributed data, and the differences between the two groups were detected using a Student’s t-test.

After incorporating the significant features in a multivariate Cox analysis, a Cox proportional-hazards model was used for survival analysis to ascertain whether GC therapy was a risk indicator for an adverse outcome in patients with SFTS. To compare in-hospital mortality between the GC and non-GC groups, Kaplan-Meier curves were created, and a log-rank test was used to statistically quantify the differences in in-hospital mortality between the two groups. We also performed sensitivity analyses in the primary and matched cohorts to further validate the conclusions of the multivariate Cox analysis.

### 2.4 Meta-analysis

The meta-analysis followed the Preferred Reporting Items for Systematic Reviews and Meta-Analyses (PRISMA), guidelines, and the protocol was registered in PROSPERO (registration number CRD42023451098) prior to commencing the literature search. Two independent reviewers (C.Y. and WH.) searched for English-language studies in PubMed, EMBASE, and the Cochrane Library. Studies were included if they met the following criteria: (i) the study design was a cohort study or a randomized controlled trial; (ii) all participants were adults and were diagnosed with SFTS based on qRT-PCR, nested PCR or metagenomics next generation sequencing to detect the serum SFTSV RNA in serum; (iii) patients’ demographic information (including gender and age) were recorded.

Review Manager Version 5.4 (The Cochrane Collaboration, 2020.) software was used to performed the statistical calculations. The risk ratios (RRs) and 95% confidence intervals (CIs) were used to present the statistical results for dichotomous outcomes. The Mantel-Haenszel analysis was adopted for dichotomous variables (Mantel and Haenszel, 1959). The level of statistical significance was set at *P*<0.05. Statistical heterogeneity was assessed based on the I-square (I²) value and was considered statistically significant at *P*<0.01. The statistical heterogeneity was classified into four categories based on the I² value: homogeneous (I²<25%), low heterogeneity (25%≤I²<50%), moderate heterogeneity (50%≤I²<75%), and high heterogeneity (I²≤75%) (Higgins and Thompson, 2002). When a study was homogeneous or had low heterogeneity (I^2^<50%), the data were pooled using the fixed-effects model. A random effects model was otherwise employed to combine the effect size, while the ***P*** value of the chi-squared (X^2^) statistic was <0.05 simultaneously (Mantel and Haenszel, 1959; DerSimonian and Laird, 1986; Higgins et al., 2003). A subgroup analysis was not performed as the meta-analysis did not have sufficient data. Potential publication bias was represented using a funnel plot. Sensitivity analysis was used to evaluate the stability of the final results of the meta-analysis (Begg and Mazumdar, 1994; Duval and Tweedie, 2000).

## 3 Results

### 3.1 Clinical features in Wuhan union cohort

In our retrospective cohort study, 566 patients diagnosed with SFTS based on PCR test between January 2020 and December 2022 were enrolled. The median age was 61.36 years. Approximately 41.70% of the enrolled patients with SFTS were males (n=236). There were 239 cases of general or mild type SFTS, and 327 severe type cases (**Supplementary Table 1**).

Patients were divided into two cohorts based on GC use. **Supplementary Table 1** outlines the characteristics of the patients, encompassing demographic details, comorbidity conditions, clinical manifestations, and a range of laboratory assessments. These laboratory assessments include complete blood counts, evaluations of liver and kidney functionality, indicators of inflammation, and the quantification of SFTS viral load. In total, 180 patients received GC therapy, and 386 did not. Those who received GC therapy in the original cohort had lower lymphocyte counts (*P*<0.001), higher lactate dehydrogenase concentrations (*P*=0.019), and higher viral loads (*P*<0.001) than those not treated with GCs. After PSM and IPTW, as shown in the matched cohort and weighted cohort, almost all covariates were balanced between the GC and non-GC group (*P*>0.05), except for the lymphocyte counts in the weighted cohort (*P*=0.002). This indicates that after our adjustments, the baseline characteristics between the two groups have approximately achieved balance.

In terms of clinical outcomes (**Supplementary Table 1**), compared to patients in the non-GC-treated group, those in the GC-treated group had higher fatality rates (21.1% vs. 11.9%, *P*=0.006) in the original cohort. Regarding the overall duration of stay, a comparable situation is noted, as patients in the original GC group had a longer length of hospital stay (10.6 ± 5.1 vs. 9.5 ± 4.2, *P*=0.033). However, in the matched and weighted cohorts, we did not observe similar evidence of increased mortality (*P*=0.422 in matched cohorts and *P*=0.114 in weighted cohorts) or length of stay (*P*=0.170 in matched cohorts and *P*=0.171 in weighted cohorts). Regarding secondary infection, neither the use of GCs nor non-GCs made a significant difference in the original (*P*=0.193), matched (*P*=0.386), and weighted cohorts (*P*=0.499).

### 3.2 Survival outcome

**Figure 1:**
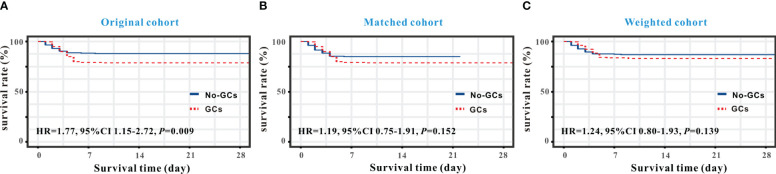
Kaplan-Meier curve analysis of survival rates between the GC and non-GC groups in SFTS individuals. Survival analysis demonstrated that GC therapy increased in-hospital mortality in the original cohort **(A)** but not in the matched cohort **(B)** and weighted cohort **(C)**.

As shown in **Figure 1**, a Kaplan-Meier curve was plotted to determine whether there was any difference in in-hospital mortality between the GC and non-GC groups. A log-rank test revealed that in the original cohort (**Figure 1A**), patients with SFTS in the GC group had a higher risk of in-hospital death [hazard ratio (HR)=1.77, 95% CI: 1.15-2.72, *P*=0.009] compared with the non-GC group. We have conducted a subgroup analysis of the original data. After separating the patients according to the severity of SFTS into General/Middle and Severe/Critically ill types, neither the duration nor the amount of GC treatment had a significant impact on the mortality risk of patients (*P*>0.05). However, we did not observe a similar higher mortality in the matched (HR= 1.19, 95% CI: 0.75-1.91, *P*=0.152, **Figure 1B**) and weighted (HR=1.24, 95% CI: 0.80-1.93, *P*=0.139) cohorts (**Figure 1C**).

**Figure 2:**
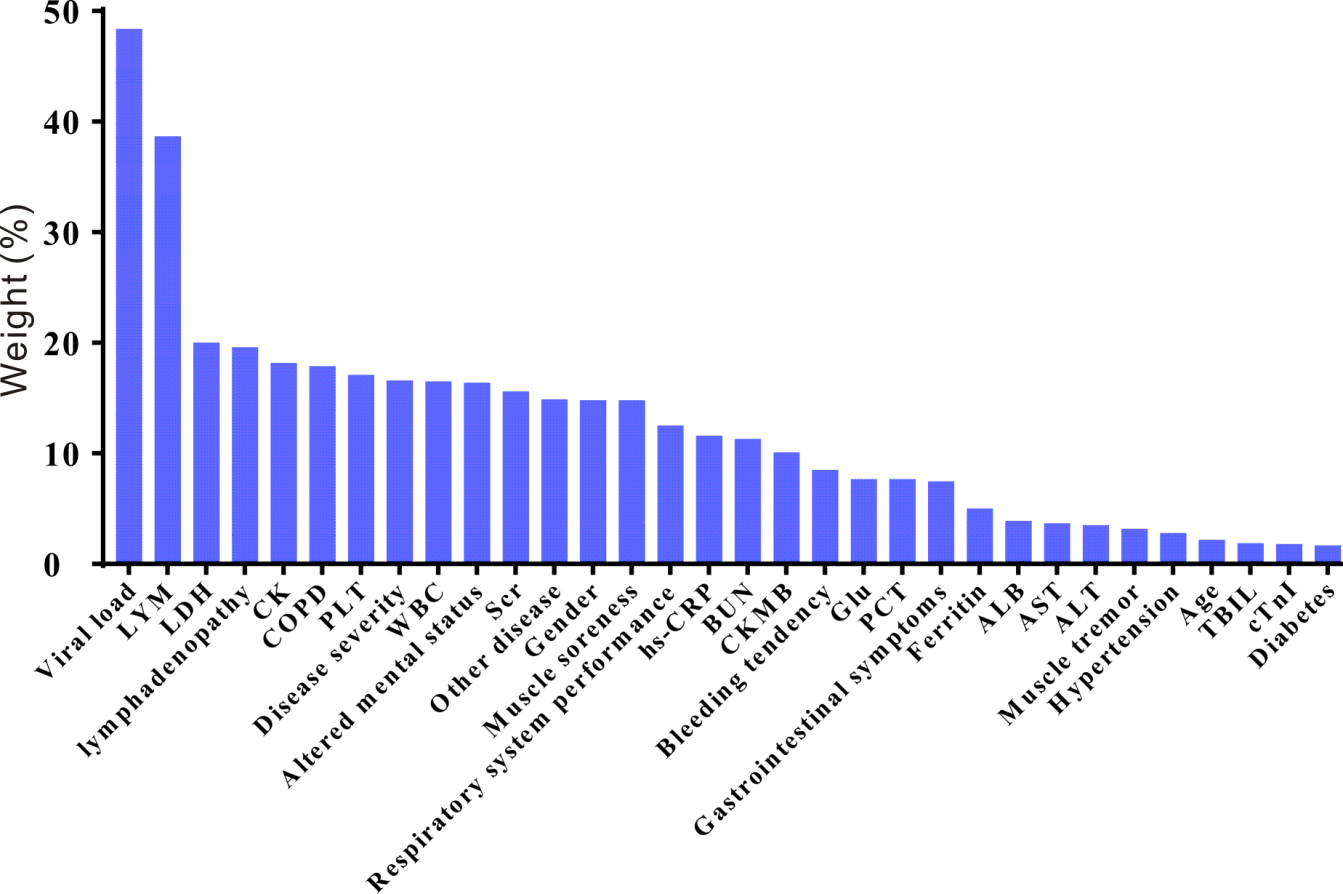
The relative risk weights of covariates in the SFTS prognostic model (ranked from high to low). LYM, lymphocyte; LDH, lactate dehydrogenase; CK, creatine kinase; COPD, chronic obstructive pulmonary disease; PLT, platelet; WBC, white blood cell; SCR, serum creatinine; hs-CRP, hypersensitive C-reactive protein; BUN, blood urea nitrogen; CKMB, creatine kinase isoenzymes; Glu, glucose; PCT, procalcitonin; ALB, albumin; AST, aspartate amino transferase; ALT, alanine aminotransferase; TBIL, total bilirubin; cTnI, cardiac troponin I.

A relative impact factor was used to measure how well the 32 covariates in the propensity score model predicted survival duration (**Figure 2**). Among them, we found that the top three clinical factors affecting the prognosis of SFTS individuals were viral load, lymphocytes and lactate dehydrogenase. While, age and some comorbids, such as hypertension and diabetes, had little impacts on the survival outcomes of SFTS individuals. We used multivariate Cox regression to explore the effects of GC on survival outcomes in the entire population, matched, and weighted cohorts (**Table 1**). After implementing a tiered adjustment for potential covariates (Adjust I model adjusted for age, gender, disease severity, viral load, and ferritin; Adjust II model adjusted for adjust I model plus comorbidities, altered mental status, bleeding tendency, gastrointestinal symptoms, respiratory system performance, muscle soreness, muscle tremor, lymphadenopathy; Adjust III model adjusted for adjust II model plus laboratory results) the association between mortality risk and the use of GC remained insignificant (*P *> 0.1).

**Table 1:** Results of multivariate cox regression of GC therapy on survival outcomes among SFTS individuals.

Methods	Overall Survival	
	HR (95% CI)	*P* value
Cox proportional hazards model	1.77 (1.15-2.72)	0.009
Cox proportional hazards model with adjust I	1.23 (1.09-2.19)	0.036
Cox proportional hazards model with adjust II	1.06 (0.85-1.67)	0.124
Cox proportional hazards model with adjust III	1.14 (0.67-1.93)	0.163
Propensity score matching	1.19 (0.75-1.91)	0.152
Propensity score matching with adjust I	1.20 (0.76-1.92)	0.146
Propensity score matching with adjust II	1.09 (0.66-1.78)	0.175
Propensity score matching with adjust III	1.02 (0.57-1.82)	0.195
Propensity score IPW	1.24 (0.80-1.93)	0.139
Propensity score IPW with adjust I	1.21 (0.78-1.88)	0.140
Propensity score IPW with adjust II	1.07 (0.67-1.70)	0.178
Propensity score IPW with adjust III	1.18 (0.69-2.03)	0.156

Adjust I model adjusted for age, gender, disease severity, viral load, and ferritin; Adjust II model adjusted for adjust I model plus comorbidities, altered mental status, bleeding tendency, gastrointestinal symptoms, respiratory system performance, muscle soreness, Muscle tremor, lymphadenopathy. Adjust III model adjusted for adjust II model plus laboratory results.

PSM, Propensity Score Matching; IPTW, inverse probability of treatment weighting.

### 3.3 Meta-analysis

**Figure 3:**
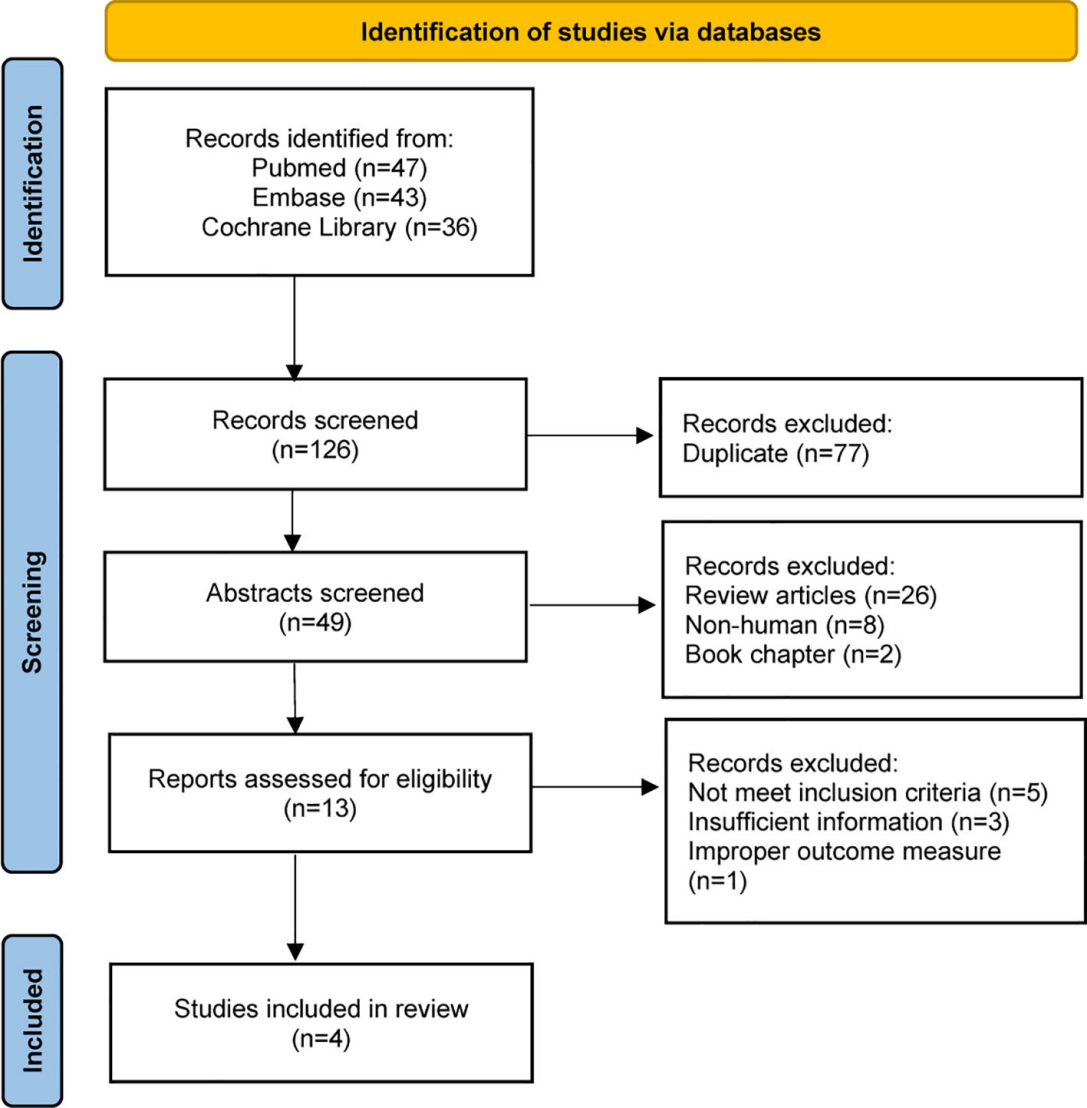
Flow chart of the study selection process in meta-analysis.

The detailed steps in the literature search are illustrated in **Figure 3**. We screened 126 references and only selected four SFTS studies according to the inclusion criteria. Four studies (Jung et al., 2021; Kawaguchi et al., 2021; Xiong et al., 2022; Wang et al., 2023) including 3111 patients and our original cohort were included in the meta-analysis. All the five clinical studies were performed in Asian countries, and three of them were from China. Among the 3677 individuals with SFTS, only 725 patients received GC therapy. Moreover, we noticed that the mean age of SFTS individuals was younger in China than Japan or Korea. The key characteristics of the SFTS patients, including sex ratio, country, age, and complication rates, are presented in **Table 2**.

**Table 2:** Patients’ characteristics of trails included in the meta-analysis.

Study	Country	Number		Female (%)		Age (year)		Duration (day)		Comorbidity (%)	
		GC	Non-GC	GC	Non-GC	GC	Non-GC	GC	Non-GC	GC	Non-GC
Jung et al (Jung et al., 2021)	Korea	58	84	50.	48.80	71.5 [62.5–77]	67 [61–73.8]	5.0 [3.0–5.3]	5.0 [3.0–6.0]	65.5	59.50
Kawaguchi et al (Kawaguchi et al., 2021)	Japan	12	12	41.7	50.00	76.5 [67.8–79.5]	78.5 [72.8–81]				
Xiong et al (Xiong et al., 2022)	China	144	323	57.6	52.90	59 [51-67]	59 [51-66]	6 [5-7]	6 [5-7]	30.6	24.80
Wang et al (Wang et al., 2023)	China	331	2147	48.9	58	67 [59-73]	62 [53-70]	5 [4-7]	5 [4-7]	46.8	42.10
Chen et al.	China	180	386	53.3	60.60	61.5 ± 11.2	61.3± 10	10.6± 5.1	9.5 ± 4.2		

Data are presented as n, n (%), median [inter-quartile range] or mean ± standard deviation. GC, glucocorticoid.

**Figure 4:**
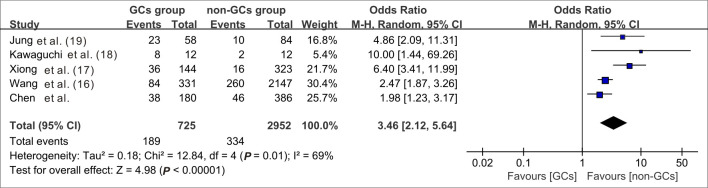
Forest plot of meta-analysis comparing the mortality rate between the GC treated and no-GC treated groups. M-H, Mantel-Haenszel; CI, confidence intervals; Tau^2^, Kendall’s tau coefficient; Chi^2^, Chi-Squared Test; df, degrees of freedom; Z, Z-score.

**Figure 5:**
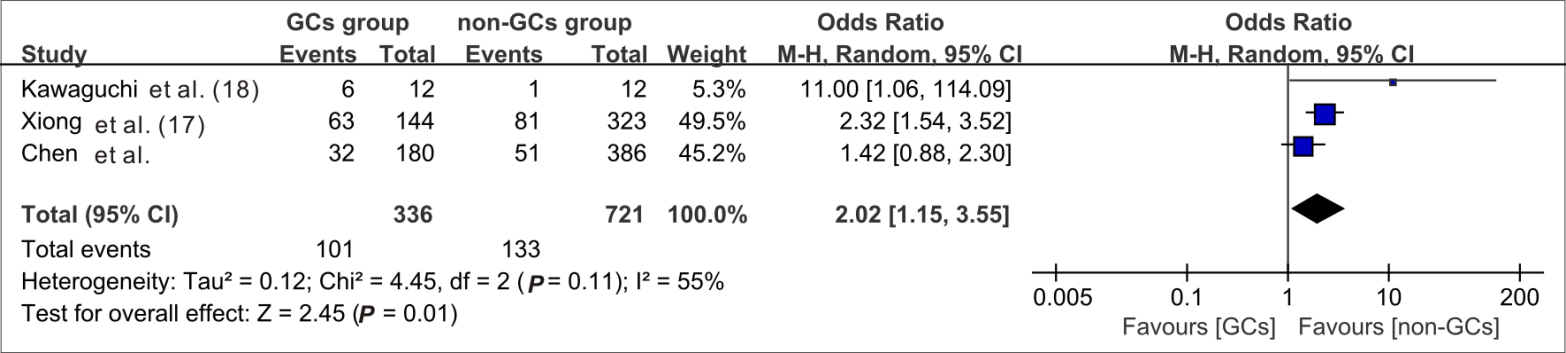
Forest plot of meta-analysis comparing the secondary infection rates between the GC treated and no-GC treated groups. M-H, Mantel-Haenszel; CI, confidence intervals; Tau^2^, Kendall’s tau coefficient; Chi^2^, Chi-Squared Test; df, degrees of freedom; Z, Z-score.

When data from the five cohort studies were combined, there was moderate heterogeneity with an I^2^ statistic of 69%, indicating that a random-effects model needs to be utilized for data analysis (**Figure 4**). The mortality rate of SFTS individuals in the GC group was 3.46 times the rate of SFTS individuals in the control group (OR=3.46, 95% CI=2.12-5.64, *P*<0.00001), and the overall difference was statistically significant. The meta-analysis based on 3677 SFTS individuals indicated that GC therapy might increase the mortality rate, and should be used with caution. Then, we assessed the second outcome of this research via meta-analysis. After combining data from two studies (Kawaguchi et al., 2021; Xiong et al., 2022) that reported secondary infection rates along with our cohorts, the infection rate in the GC group was statistically greater than in the non-GC group (OR=1.97, 95% CI=1.45 to 2.67, *P <*0.0001) (**Figure 5**). The meta-analysis based on three studies indicated that GC therapy might increase the risk of secondary infection and should be used with caution in the clinical practice. Given the included studies provided insufficient data for analyzing a dissymmetric funnel (Sutton et al., 2000), publication bias assessment could not be utilized to evaluate the conclusions of the two meta-analyses.

## 4 Discussion

SFTS is an emerging infectious disease identified in these 15 years, resulting in a reduction of the mortality rate from an initial 30% (Yu et al., 2011) to 5.11% today (Chen et al., 2022). With a deeper understanding of the pathophysiological processes in SFTS, clinicians have drawn inspiration from the treatment of other life-threatening infectious diseases to significantly expand the therapeutic choices for treating SFTS. In addition to empiric antivirals and supportive therapy, it is undeniable that the prudent GC use for suppressing cytokine storms contributes to the reduction of mortality to some extent in clinical practice. Similarly, GC (especially dexamethasone) can reduce the mortality rate among severe and critically ill patients with COVID-19, with no increasing significant effect on the incidence of adverse reactions (de Lemos Neto et al., 2021; Qiao et al., 2023).

However, four previous retrospective cohort studies, including our own study (**Supplementary Table 1**), show a statistically significant greater mortality rate in patients administered GC therapy, and meta-analyses validated this conclusion (**Figure 4**). To adjust for baseline differences between the GC and non-GC groups, one-to-one PSM and an IPTW algorithm were employed (**Supplementary Table 1**), with outcomes indicating that whether GCs were applied or not did not affect mortality rates. In terms of survival time, while patients using GC had a shorter in-hospital survival time than those in the non-GC group, after adjustment using PSM and the IPTW algorithm, no between-group difference was observed in terms of mortality risk (**Figure 1**). We considered that the major cause of higher mortality rate in the GC-applied group was the baseline imbalance between the two groups. In previous retrospective clinical studies (Ding et al., 2014; Zu et al., 2022; Chen et al., 2023), a higher risk of death was observed in patients with SFTS with advanced age, comorbidities or underlying disorders, and elevated D-dimer levels. Such patients had a higher probability of developing a very severe form; a higher probability of developing a cytokine storm; a higher probability of complications such as myocarditis, encephalitis and hemophagocytic syndrome; and a higher probability of GC use in clinical practice. However, none of the therapies could guarantee that patients with a very severe form or who were critically ill would survive or have a lower death rate than patients with the general type. Thus, without modifying the baseline characteristics, the initial cohort was unable to demonstrate the negative effects of GC therapy on mortality outcomes (**Supplementary Table 1**). We specifically used multivariate Cox regression for adjustment in the initial cohort, and after further adjustments for comorbidities, altered mental status, bleeding tendency, gastrointestinal symptoms, respiratory system performance, muscle soreness, muscle tremor, and lymphadenopathy based on age, sex, disease severity, viral load, and ferritin levels, no statistically significant difference in survival time between the GC and non-GC groups was identified (**Table 1**).

Extended use of GC or broad-spectrum antibiotics increases the risk of developing secondary bacterial and fungal infections. Xu et al (Xu et al., 2021). showed that invasive pulmonary aspergillosis (IPA) was present in 29 (31.9%) of 91 patients with SFTS. SFTS and IPA are associated with significant morbidity and mortality rates; therefore, we focused on the secondary infection rate following GC therapy. Our retrospective cohort study findings showed no significant differences in secondary infection rates between the GC and non-GC groups, irrespective of baseline adjustments (**Supplementary Table 1**). However, the GC group had a higher incidence of subsequent infections when combined with data from two previous trials (Kawaguchi et al., 2021; Xiong et al., 2022) in meta-analysis, but our meta-analysis findings are limited (**Figure 5**), as the baseline data from previous studies was not balanced. Both our study and that of Xiong et al. research (Xiong et al., 2022) were conducted at the same large medical institution. Our cohort had attended the hospital later than theirs, and the two retrospective cohorts did not overlap in time. However, the conclusions were contrary to ours but compatible with the meta-analysis pooled results. We speculate that this contradictory situation resulted from our increasing experience in treating patients with SFTS over time. In our practice, we have empirically used GC therapy in conjunction with antibiotics to treat hospital-acquired pneumonia. Furthermore, we conducted serum G and GM tests, which detect distinctive components of the fungal cell wall, and administered antifungal medications in cases where fungal infection was suspected. Consequently, the incidence of secondary infections in our retrospective study from 2020 to 2022 was not significantly different between the GC and non-GC groups (**Supplementary Table 1**). One reason for the better therapeutic effects of GCs in COVID-19 versus SFTS may be attributed to the fact that COVID-19 predominantly targets the respiratory system and consequently, clinicians often prescribe antibiotics on an empirical basis to mitigate the risk of secondary bacterial infections by overly immunocompromised during the viral infection. Unfortunately, there are still no guidelines specifying when and which antibacterial drugs should be used, leaving the risk of secondary infections in patients with SFTS entirely dependent on the clinician’s personal experience.

This is the first meta-analysis to investigate the effect of GC therapy on death and secondary infection rates in patients with SFTS. Our sample size of SFTS patients in the retrospective cohorts was 566, establishing it as one of the largest among published studies. Furthermore, our institution, Wuhan Union Hospital, was among the earliest facilities to identify SFTS (Zhang et al., 2012) and has abundant experience in treating this emerging infectious disease. Therefore, the adequate sample size of patients with SFTS in our institution provides a strong basis for our reliable conclusions.

Our study had some limitations. First, this was a single center study, involving patients from Wuhan Union Hospital, which is also the hospital used in the research by Xiong et al (Xiong et al., 2022). This prevented us from acquiring external validation for our retrospective cohort study, thus, reducing the reliability of the meta-analysis to some extent. Second, our retrospective investigation did not provide sufficiently clear outcomes, which limits understanding of the effects of GC therapy. Moreover, different GCs and GC dosages were not thoroughly evaluated, making our study inadequate for establishing clinical guidelines. More studies on the use of GC therapy in patients with SFTS, especially in very severe SFTS cases, is anticipated, which may help to inform evidence-based medical recommendations for the clinical application of GC in SFTS patients. Further research is needed to explore the effects of various GC types, dosages, usage patterns, and treatment durations in patients with SFTS, which will help improve evidence-based medical recommendations for the clinical application of GC in patients with SFTS.

## 5 Conclusion

GC therapy should be used with caution when treating patients with SFTS, despite no significant change in mortality or secondary infection risk among such patients. In patients with severe cases, GC remains an option; however, caution must be exercised to prevent secondary infections, and antibiotics and antifungals should be used empirically if necessary.

## Data availability statement

The original contributions presented in the study are included in the article/**Supplementary Material**. Further inquiries can be directed to the corresponding authors.

## Ethics statement

The studies involving humans were approved by the institutional review board of Tongji Medical College (2023-S093). The studies were conducted in accordance with the local legislation and institutional requirements. The ethics committee/institutional review board waived the requirement of written informed consent for participation from the participants or the participants’ legal guardians/next of kin because this is a retrospective clinical research.

## Author contributions

CG: Conceptualization, Investigation, Supervision, Visualization, Writing – review & editing. YC: Formal analysis, Investigation, Methodology, Writing – original draft. HW: Data curation, Methodology, Project administration, Software, Writing – review & editing. FZ: Investigation, Methodology, Supervision, Validation, Writing – review & editing.

## Supplementary material

Supplementary Table 1 Comparisons of baseline characteristics between the original cohort, matched cohort and weighted cohort.
