# Supplementary material for: The use of glucocorticoid in severe fever with thrombocytopenia syndrome: a retrospective cohort study
Source: Front Cell Infect Microbiol. 2024 Aug 6;14:1419015. doi: 10.3389/fcimb.2024.1419015 (PMC11333439; doi:10.3389/fcimb.2024.1419015)
Supplement: Supplementary Table 1 — Comparisons of baseline characteristics between the original cohort, matched cohort and weighted cohort. [file Table_1.docx]

Supplementary Table 1. Comparisons of baseline characteristics between the original cohort, matched cohort and weighted cohort

| Covariates | Original cohort | | | Matched cohort | | | Weighted cohort | | |
| --- | --- | --- | --- | --- | --- | --- | --- | --- | --- |
|  | No-GCs | GCs | P value | No-GCs | GCs | P value | No-GCs | GCs | P value |
| N | 386 | 180 |  | 180 | 180 |  | 179.9 | 180.0 |  |
| Age, years old | 61.3 ± 10.2 | 61.5 ± 11.2 | 0.807 | 62.0 ± 10.2 | 61.5 ± 11.2 | 0.676 | 61.4 ± 10.2 | 61.5 ± 11.2 | 0.930 |
| Gender, male, n (%) | 152 (39.4) | 84 (46.7) | 0.122 | 82 (45.6) | 84 (46.7) | 0.916 | 83.4 (46.3) | 84 (46.7) | 0.943 |
| Disease severity, n (%) |  |  | 0.082 |  |  | 0.826 |  |  | 0.971 |
| General/middle | 173 (44.8) | 66 (36.7) |  | 63 (35.0) | 66 (36.7) |  | 65.6 (36.5) | 66.0 (36.7) |  |
| Severe | 213 (55.2) | 114 (63.3) |  | 117 (65.0) | 114 (63.3) |  | 114.3 (63.5) | 114.0 (63.3) |  |
| Comorbidities, n (%) |  |  |  |  |  |  |  |  |  |
| Hypertension | 94 (24.4) | 46 (25.6) | 0.838 | 45 (25.0) | 46 (25.6) | 1.000 | 42.2 (23.4) | 46 (25.6) | 0.596 |
| Diabetes | 34 (8.8) | 15 (8.3) | 0.979 | 15 (8.3) | 15 (8.3) | 1.000 | 15.9 (8.8) | 15 (8.3) | 0.857 |
| COPD | 22 (5.7) | 4 (2.2) | 0.104 | 10 (5.6) | 4 (2.2) | 0.173 | 9.8 (5.4) | 4 (2.2) | 0.088 |
| Other diseases | 67 (17.4) | 42 (23.3) | 0.118 | 34 (18.9) | 42 (23.3) | 0.366 | 31.8 (17.7) | 42 (23.3) | 0.134 |
| Altered mental status, n (%) | 114 (29.5) | 67 (37.2) | 0.084 | 69 (38.3) | 67 (37.2) | 0.913 | 64.7 (36.0) | 67 (37.2) | 0.782 |
| Bleeding tendency, n (%) | 73 (18.9) | 31 (17.2) | 0.700 | 39 (21.7) | 31 (17.2) | 0.334 | 38.9 (21.6) | 31 (17.2) | 0.280 |
| Gastrointestinal symptoms, n (%) | 313 (81.1) | 145 (80.6) | 0.774 | 148 (82.2) | 145 (80.6) | 0.787 | 149.7 (83.2) | 145 (80.6) | 0.465 |
| Respiratory system performance, n (%) | 115 (29.8) | 48 (26.7) | 0.262 | 58 (32.2) | 48 (26.7) | 0.322 | 58.1 (32.3) | 48 (26.7) | 0.283 |
| Muscle soreness, n (%) | 125 (32.4) | 71 (39.4) | 0.121 | 70 (38.9) | 71 (39.4) | 1.000 | 64.2 (35.7) | 71 (39.4) | 0.411 |
| Muscle tremor, n (%) | 58 (15.0) | 25 (13.9) | 0.819 | 35 (19.4) | 25 (13.9) | 0.203 | 33.4 (18.6) | 25 (13.9) | 0.192 |
| lymphadenopathy, n (%) | 72 (18.7) | 21 (11.7) | 0.049 | 34 (18.9) | 21 (11.7) | 0.079 | 34.8 (19.3) | 21 (11.7) | 0.213 |
| Laboratory results |  |  |  |  |  |  |  |  |  |
| WBC | 3.7 ± 1.8 | 3.3 ± 1.6 | 0.075 | 3.7 ± 1.7 | 3.3 ± 1.6 | 0.135 | 3.6 ± 1.8 | 3.3 ± 1.6 | 0.114 |
| PLT | 50.7 ± 22.5 | 47.0 ± 20.5 | 0.064 | 46.2 ± 20.8 | 47.0 ± 20.5 | 0.705 | 46.8 ± 21.2 | 47.0 ± 20.5 | 0.890 |
| LYM | 0.9 ± 0.4 | 0.7 ± 0.3 | <0.001* | 0.8 ± 0.3 | 0.7 ± 0.3 | 0.060 | 0.9 ± 0.4 | 0.7 ± 0.3 | 0.002* |
| AST | 334.1 ± 74.6 | 350.6 ± 92.2 | 0.698 | 431.9 ± 95.6 | 350.6 ± 92.2 | 0.156 | 379.9 ± 63.6 | 350.6 ± 92.2 | 0.508 |
| ALT | 124.7 ± 47.3 | 119.7 ± 36.3 | 0.707 | 151.1 ± 34.4 | 119.7 ± 36.3 | 0.078 | 133.8 ± 46.3 | 119.7 ± 36.3 | 0.307 |
| TBIL | 11.3 ± 5.0 | 11.5 ± 5.9 | 0.829 | 11.5 ± 5.9 | 11.5 ± 5.9 | 0.981 | 11.7 ± 5.5 | 11.5 ± 5.9 | 0.759 |
| ALB | 31.5 ± 4.5 | 31.7 ± 4.5 | 0.666 | 30.9 ± 4.5 | 31.7 ± 4.5 | 0.102 | 31.0 ± 4.5 | 31.7 ± 4.5 | 0.116 |
| SCR | 78.3 ± 27.8 | 85.8 ± 36.2 | 0.087 | 81.2 ± 35.3 | 85.8 ± 36.2 | 0.345 | 82.4 ± 31.2 | 85.8 ± 36.2 | 0.458 |
| BUN | 5.5 ± 1.6 | 5.9 ± 1.9 | 0.216 | 5.7 ± 1.8 | 5.9 ± 1.9 | 0.624 | 5.9 ± 1.9 | 5.9 ± 1.9 | 0.898 |
| LDH | 1028.6 ± 399.5 | 1267.5 ± 561.2 | 0.019* | 1204.9 ± 67.2 | 1267.5 ± 561.2 | 0.628 | 1168.6 ± 315.5 | 1267.5 ± 561.2 | 0.417 |
| CK | 1127.8 ± 437.5 | 1652.9 ± 584.9 | 0.858 | 1313.1 ± 573.6 | 1652.9 ± 584.9 | 0.278 | 1376.6 ± 418.2 | 1652.9 ± 584.9 | 0.363 |
| CKMB | 90.6 ± 21.8 | 118.9 ± 40.3 | 0.231 | 85.3 ± 39.3 | 118.9 ± 40.3 | 0.291 | 97.7 ± 26.1 | 118.9 ± 40.3 | 0.477 |
| cTnI | 507.4 ± 121.8 | 444.8 ± 159.9 | 0.858 | 817.4 ± 166.2 | 444.8 ± 159.9 | 0.462 | 644.2 ± 151.5 | 444.8 ± 159.9 | 0.547 |
| Ferritin |  |  | 0.672 |  |  | 1.000 |  |  | 0.993 |
| ≥ 10000 | 56 (14.5) | 23 (12.8) |  | 22 (12.2) | 23 (12.8) |  | 23.0 (12.8) | 23 (12.8) |  |
| <10000 | 330 (85.5) | 157 (87.2) |  | 158 (87.8) | 157 (87.2) |  | 156.9 (87.1) | 157 (87.2) |  |
| Glu | 7.2 ± 3.1 | 7.4 ± 2.9 | 0.398 | 7.4 ± 2.6 | 7.4 ± 2.9 | 0.937 | 7.4 ± 3.2 | 7.4 ± 2.9 | 0.979 |
| Viral load |  |  | <0.001* |  |  | 1.000 |  |  | 1.000 |
| ≥ 1000 | 176 (45.6) | 124 (68.9) |  | 124 (68.9) | 124 (68.9) |  | 124.0 (68.9) | 124 (68.9) |  |
| <1000 | 210 (54.4) | 56 (31.1) |  | 56 (31.1) | 56 (31.1) |  | 55.9 (31.1) | 56 (31.1) |  |
| hs-CRP | 9.8 ± 3.1 | 11.8 ± 5.2 | 0.179 | 10.5 ± 5.2 | 11.8 ± 5.2 | 0.476 | 9.8 ± 3.4 | 11.8 ± 5.2 | 0.234 |
| PCT | 0.8 ± 0.2 | 1.1 ± 0.5 | 0.376 | 1.2 ± 0.5 | 1.1 ± 0.5 | 0.720 | 0.9 ± 0.3 | 1.1 ± 0.5 | 0.546 |
| Outcomes |  |  |  |  |  |  |  |  |  |
| Time | 9.5 ± 4.2 | 10.6 ± 5.1 | 0.033* | 9.7 ± 5.0 | 10.6 ± 5.1 | 0.170 | 9.8 ± 3.9 | 10.6 ± 5.1 | 0.171 |
| death, n (%) | 46 (11.9) | 38 (21.1) | 0.006* | 31 (17.2) | 38 (21.1) | 0.422 | 27.6 (15.3) | 38 (21.1) | 0.114 |
| Secondary infection, n (%) | 51 (13.2) | 32 (17.8) | 0.193 | 35 (13.9) | 32 (17.8) | 0.386 | 27.6 (15.4) | 32 (17.8) | 0.499 |

Data are presented as n, n (%) or mean ± standard deviation.

*：There is a statistically significant difference.

Abbreviations: COPD, chronic obstructive pulmonary disease; WBC, white blood cell; PLT, platelet; LYM, lymphocyte; AST, aspartate amino transferase; ALT, alanine aminotransferase; TBIL, total bilirubin; ALB, albumin; SCR, serum creatinine; BUN, blood urea nitrogen; LDH, lactate dehydrogenase; CK, creatine kinase; CKMB, creatine kinase isoenzymes; cTnI, cardiac troponin I; Glu, glucose; hs-CRP, hypersensitive C-reactive protein; PCT, procalcitonin.
